# Left portal vein thrombosis as an unusual complication following acute cholecystitis: case report and literature review

**DOI:** 10.1093/jscr/rjaf118

**Published:** 2025-03-07

**Authors:** Faisal A Almudaiheem, Mohammed Aljabali, Bandar Ali, Majed Alanazi

**Affiliations:** Department of General Surgery, Prince Sultan Military Medical City, Makkah Al Mukarramah Road, As Sulimaniyah District, Riyadh, 12233, Saudi Arabia; Department of General Surgery, Prince Sultan Military Medical City, Makkah Al Mukarramah Road, As Sulimaniyah District, Riyadh, 12233, Saudi Arabia; Department of General Surgery, Prince Sultan Military Medical City, Makkah Al Mukarramah Road, As Sulimaniyah District, Riyadh, 12233, Saudi Arabia; Department of General Surgery, Prince Sultan Military Medical City, Makkah Al Mukarramah Road, As Sulimaniyah District, Riyadh, 12233, Saudi Arabia

**Keywords:** acute cholecystitis, portal vein thrombosis, laparoscopic cholecystectomy

## Abstract

Portal vein thrombosis (PVT) is a rare but potentially severe condition that is typically associated with underlying haematological disorders, genetic mutations, or liver diseases such as cirrhosis. However, PVT resulting from acute cholecystitis is an exceedingly uncommon occurrence with few documented cases. This report describes the case of a 44-year-old man who presented with acute right upper quadrant pain and was diagnosed with acute cholecystitis complicated by left-sided PVT, which was managed with anticoagulants and laparoscopic cholecystectomy. This case underscores the complexity of managing acute cholecystitis complicated by PVT. Although anticoagulation remains the cornerstone of PVT management, concurrent surgical intervention for cholecystitis poses challenges in terms of timing and perioperative care. A multidisciplinary approach tailored to the individual patient is crucial to achieving optimal outcomes.

## Introduction

Portal vein thrombosis (PVT) is considered a rare disease, occurring in only 1% of the general population, with the most common causes being haematological disorders, genetic mutations, or liver cirrhosis [1]. The benefits of early diagnosis and management have been thoroughly reported, as delays in management can lead to catastrophic complications [2–4]. Development of PVT as a result of acute cholecystitis is rare, with few cases documented in the literature, each managed differently. Herein, we report a case of acute cholecystitis complicated by left-sided PVT.

## Case report

A 44-year-old Saudi male presented to our emergency department with a history of persistent right upper quadrant pain that started two days prior. The pain was sharp in nature, radiating to the right shoulder, with no aggravating factors and temporarily relieved by analgesia; it was associated with nausea, subjective fever, and a history of dark urine. He denied any other symptoms and had an unremarkable systemic review. On physical examination, his vital signs were within normal ranges. His abdomen was positive for right upper quadrant pain and a positive Murphy’s sign; otherwise, his examination was unremarkable. Laboratory results are shown in Table 1.

**Table 1:** Laboratory results upon presentation

**Test name**	**Test result**
White blood cell	28.70 10^9^/L (high)
Haemoglobin	133.0 g/L
Platelets	174 10^9^/L
International normalization ratio	1.7 (High)
Creatinine	88 mcmol/L
Blood urea nitrogen (BUN)	3.7 mmol/L
Bilirubin total	28.0 mcmol/L (high)
Bilirubin direct	14.7 mcmol/L (high)
Alanine aminotransferase	52 unit/L (high)
Aspartate aminotransferase	45 unit/L (high)
Alkaline phosphatase	78 unit/L
Sodium	131 mmol/L (low)
Potassium	3.4 mmol/L (low)
Amylase level	34 unit/L

**Figure 1:**
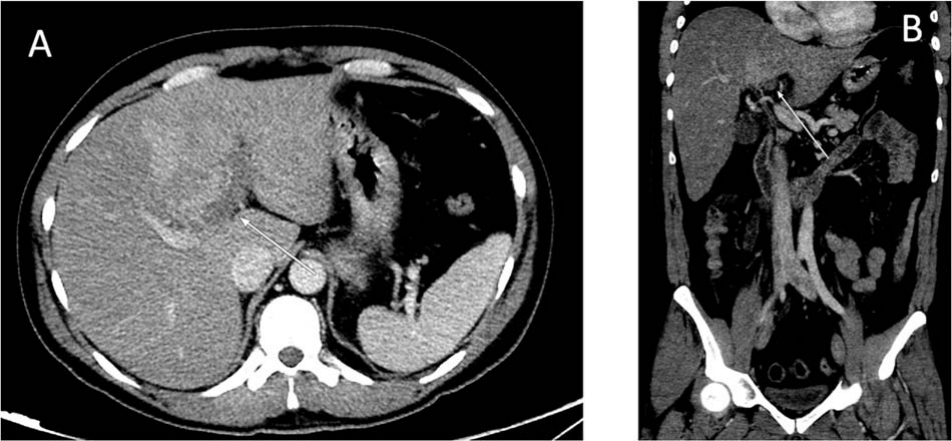
Contrast-enhanced CT scan of the abdomen: (A) axial cut; (B) coronal cut. Arrow indicates left portal vein thrombosis.

**Figure 2:**
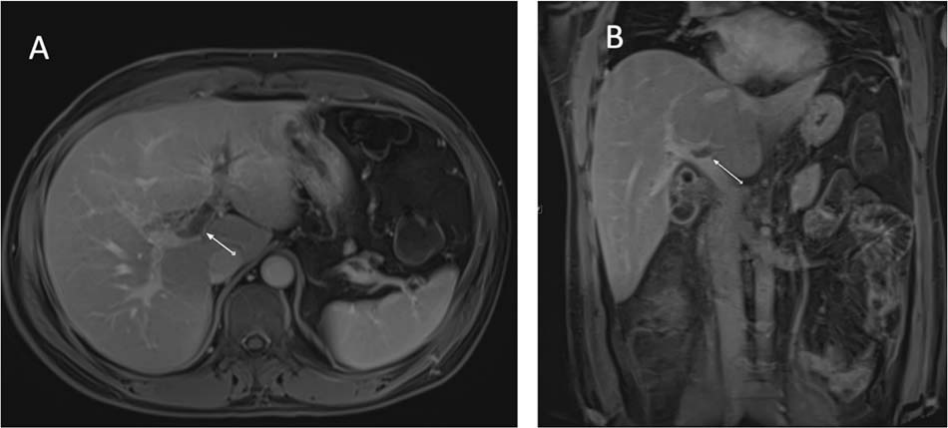
MRCP T1-weighted images: (A) axial cut; (B) coronal cut. Arrow indicates left portal vein thrombosis.

**Figure 3:**
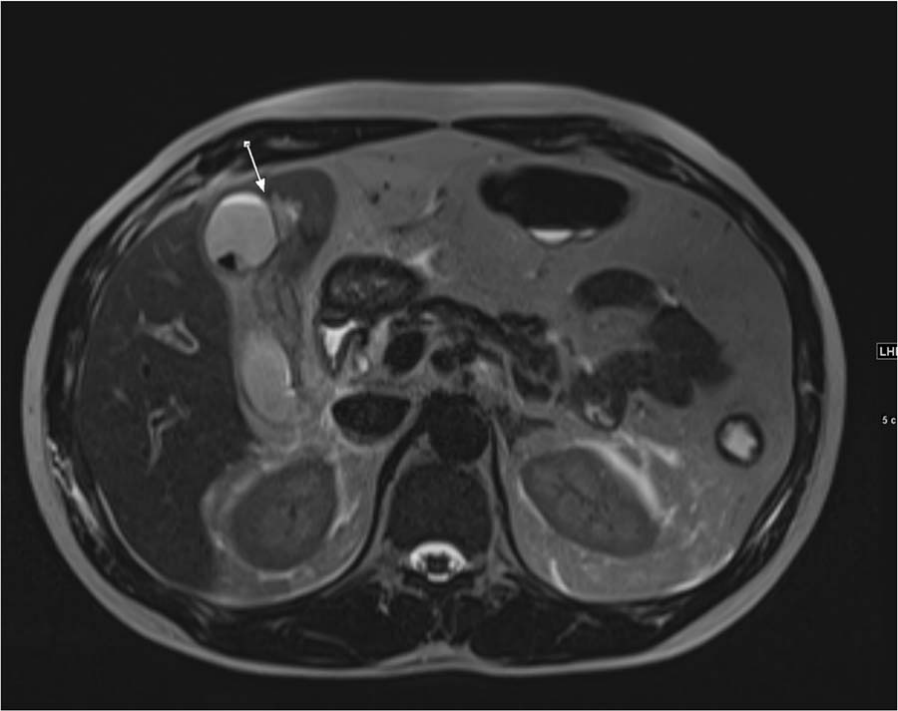
Axial cut MRCP showing T2-weighted images. Arrow indicates area of micro-perforation and small collection.

An ultrasound of the abdomen showed an impacted stone at the neck of the gallbladder with acute cholecystitis. The patient was admitted and started on IV antibiotics. He developed tachycardia reaching 110 bpm and had a high white blood cell count; therefore, the decision was made to proceed with a contrast-enhanced computed tomography (CT) scan, which showed evidence of acute cholecystitis and left PVT (Fig. 1). The medical team was consulted, and IV heparin without bolus was started. The gastroenterology team was consulted for his high bilirubin levels and advised for magnetic resonance cholangiopancreatography (MRCP), which was done the next day and confirmed the findings of left PVT (Fig. 2). MRCP also showed a micro perforation of the gallbladder and no biliary obstruction or stones (Fig. 3).

The patient was then taken for laparoscopic cholecystectomy. Intraoperatively, gangrenous cholecystitis was found, along with a pus collection in the gallbladder bed, which was opened and evacuated. A completion cholecystectomy was performed, and a Jackson-Pratt drain was placed in the area of concern. Postoperatively, the patient began recovering well: his laboratory results normalized, all septic workup was negative, and the drain exhibited minimal haemoserous fluids, which was removed on postoperative day 4. The patient was discharged the following day in good, stable condition.

At a follow-up appointment in the outpatient clinic two weeks later, the patient was doing well with no active complaints, and he was compliant with his enoxaparin regimen as instructed. His physical exam showed healed surgical wounds, and his laboratory workup was within normal ranges. Pathology results indicated gangrenous cholecystitis. The patient was then discharged and continued follow-up in the haematology clinic.

## Discussion

This case report highlights a rare instance of PVT arising as a complication of acute cholecystitis. The occurrence of PVT in association with acute cholecystitis is uncommon but has been documented in case reports and small case series, with broad variations in management [5–9]. The pathogenesis of acute cholecystitis is mainly due to obstructed biliary outflow [10], and it is typically diagnosed with ultrasound, whereas CT is reserved for uncertain or suspected complicated cholecystitis [11]. PVT often remains undiagnosed until it is detected incidentally during examination for other reasons.

As mentioned previously, the pathogenesis of PVT is multifactorial, with contrast-enhanced CT able to diagnose 90% of cases [12]. In the present case, local causes of PVT, such as trauma, acute pancreatitis, diverticulitis, cholangitis, or previous abdominal surgery [2], were not observed. However, the initial triggering factor for PVT in this case may have been the intense inflammatory response caused by the stone in the cystic duct, which is in close proximity to the draining cystic veins in Calot’s triangle [13].

Presentation of PVT can vary significantly, but the most common symptoms include abdominal pain, nausea, vomiting, and diarrhoea. Patients often present with non-specific signs such as abdominal tenderness, distension, reduced bowel sounds, fever, and shock [14]. The main treatment for PVT is anticoagulation, with the dual aims of preventing thrombus expansion and promoting natural recanalization [15]. In contrast, laparoscopic cholecystectomy is the gold standard for management of acute cholecystitis [11]. Therefore, having both diseases simultaneously presents a dilemma for the surgeon. Successful conservative management of acute cholecystic with antibiotics and hydration has been reported [6–8]. Menéndez-Sánchez *et al.* [16] reported a case of acute septic PVT requiring source control *via* laparoscopic cholecystectomy and antibiotics, but no anticoagulation was started. In contrast, it is feasible and safe to start PVT treatment with anticoagulants, and to proceed with laparoscopic cholecystectomy with appropriate perioperative anticoagulant precautions.

The coexistence of PVT and acute cholecystitis poses significant diagnostic and therapeutic challenges, particularly in determining the appropriate timing and approach to management. Although anticoagulation remains the cornerstone of PVT treatment, the concurrent management of acute cholecystitis through laparoscopic cholecystectomy requires careful consideration of perioperative anticoagulation strategies. This report highlights the need for individualized, multidisciplinary approaches to optimize outcomes in such rare and challenging cases.
